# Clinical rationale behind glucose-sparing strategies in peritoneal dialysis: a narrative review

**DOI:** 10.1186/s12882-025-04732-w

**Published:** 2026-01-13

**Authors:** Bengt Lindholm, Antoine Barbari, Jennifer Allen, Inès Dufour, Donald Fraser, Annette Heider, Rumeyza Kazancioglu, Monika Lichodziejewska-Niemierko, Anabela Malho-Guedes, Loris Neri, Alena Parikova, Juan Carlos Quevedo-Reina, Adonay Santana-Quintana, Udaya Udayaraj

**Affiliations:** 1 Renal Medicine, Karolinska Institutet, M99 Karolinska University Hospital Huddinge, Stockholm, 14186 Sweden; 2 Faculty of Medical Sciences, Lebanese University, Beirut, Lebanon; 3 Renal and Transplant Unit, Nottingham University Hospitals NHS Trust, Nottingham, UK; 4 Division of Nephrology, Cliniques Universitaires Saint-Luc, Brussels, Belgium; 5 Wales Kidney Research Unit, School of Medicine, College of Biological and Life Sciences, Cardiff University, Cardiff, UK; 6 Department of Nephrology, Klinikum Neumarkt and KfH Neumarkt, Neumarkt, Germany; 7 Division of Nephrology, Bezmialem Vakif University Faculty of Medicine, Istanbul, Türkiye; 8 Department of Nephrology Transplantology and Internal Medicine, Medical University Hospital of Gdańsk, Gdańsk, Poland; 9 Department of Palliative Medicine, Medical University of Gdańsk, Gdańsk, Poland; 10 Serviço de Nefrologia, Unidade Local de Saúde do Algarve, Faro, Portugal; 11 Renal and Dialysis Unit, Michele e Pietro Ferrero Hospital, Verduno, CN Italy; 12 Department of Nephrology, Transplant Center, Institute for Clinical and Experimental Medicine, Prague, Czech Republic; 13 Servicio de Nefrología, Hospital Universitario de Gran Canaria Doctor Negrín, Las Palmas, Spain; 14 Oxford Kidney Unit, Churchill Hospital, Oxford, UK; 15 Nuffield Department of Medicine, University of Oxford, Oxford, UK

**Keywords:** Glucose sparing, Peritoneal dialysis, Clinical outcomes, Adverse events, PD solutions

## Abstract

The effectiveness of peritoneal dialysis (PD) relies on dialysate-induced solute and water transport across the peritoneal membrane, facilitated by concentration and type of osmotic agents. Standard PD solutions predominantly use glucose as an osmotic agent due to its well-known metabolism, effective ultrafiltration during shorter dwells, and low cost. However, glucose exposure may damage the structure and function of the peritoneal membrane and cause systemic metabolic complications, including insulin resistance and cardiovascular disease, underscoring the need for glucose-sparing strategies with alternative solutions, such as solutions with icodextrin and amino acids as osmotic agents, and glucose-based, less bioincompatible fluids with physiological pH and reduced glucose degradation products. This brief narrative review examines the unwanted effects of glucose-based solutions and the clinical rationales behind glucose-sparing strategies that may reduce these effects and potentially improve clinical outcomes.

## Background

End-stage kidney disease (ESKD) necessitating kidney replacement therapies such as kidney transplantation, hemodialysis (HD), or peritoneal dialysis (PD) represents a significant global health burden [1]. Among these, PD is a well-established, cost-effective modality with notable advantages over HD, including greater preservation of residual kidney function (RKF), improved quality of life, and lower dependency on healthcare infrastructure [2–5]. Unlike HD, which requires extracorporeal blood circulation through vascular access, PD employs the peritoneal membrane (PM) for solute and fluid exchange, and like home HD, PD allows patients to undergo dialysis at home, which leads to greater independence and flexibility [6, 7]. The effectiveness of PD relies on dialysate-induced solute and water transport across the peritoneal membrane, facilitated by concentration and type of osmotic agents [8–10]. Standard PD solutions predominantly use glucose as an osmotic agent due to its high efficiency and low cost [4, 7–12]. However, prolonged exposure to high glucose solutions poses significant risks, including the formation of glucose degradation products (GDP), structural and functional PM damage, and systemic metabolic complications, including insulin resistance and cardiovascular disease [12–18]. The risk of the rare but catastrophic complication of PD in the form of encapsulating peritoneal sclerosis has also been associated with higher glucose exposure and GDPs [19]. These adverse effects underscore the need for innovative glucose-sparing strategies.

The bioincompatibility of conventional glucose-based dialysis fluid has driven the development of alternative solutions, such as glucose free fluids with icodextrin and amino acids as osmotic agents, and glucose-based biocompatible fluids with neutral or physiological pH and reduced GDPs. Icodextrin, a glucose polymer derived from corn starch, offers iso-osmolar properties without glucose-induced toxicity. Studies highlight its ability to mitigate risks of ultrafiltration (UF) failure, cardiovascular events, and RKF decline, particularly in patients with glucose intolerance [7, 20–23]. Other advantages include increased flexibility in schedules due to the long dwell times, which is of importance for the two increasingly common modalities, assisted PD and incremental PD. However, although well-tolerated by most patients, an increased incidence of skin rashes has been reported with icodextrin.

Current practice limits icodextrin to a single daily fluid exchange, and most patients thus remain dependent on glucose-based solutions for many daily exchanges of dialysis fluid. However, while the manufacturer’s recommendation is for one icodextrin exchange per day, off-label use of two icodextrin exchanges per day is practiced and reported to be viable [24].

Another approach to limit PM damage due to glucose toxicity is the use of multi-compartment PD solutions. These enable the separation of glucose and buffer components during sterilization, reducing GDP formation and minimizing PM damage [7, 20–23, 25–27].

Despite these advancements, long-term PD and the cumulative PM exposure to hyperosmolar glucose-based solutions often lead to structural changes, including peritoneal fibrosis and functional changes such as increasing peritoneal solute transfer rate (PSTR) and insufficient UF capacity [28].

Additionally, complications such as loss of residual kidney function, peritonitis and patient fatigue contribute to the eventual transition from PD to HD in many cases [3, 7–11, 18, 20–23, 25–29]. While glucose-sparing PD solutions, and other glucose-sparing strategies such as pharmacological therapies are promising in reducing such complications, their cost and uncertain impact on long-term survival necessitate further investigation [7, 10, 12, 20, 21].

This narrative review examines the clinical rationale behind glucose-sparing strategies in PD. By analyzing improvements in dialysate formulations and their implications for PM preservation as well as the reduction of metabolic and cardiovascular risk factors, we aim to highlight the potential of these and other glucose-sparing innovations to enhance patient outcomes and address persistent challenges in PD therapy.

## Methods

### Search strategy and eligibility criteria

A comprehensive non-systematic review of the literature was conducted in August 2025, covering all relevant publications indexed in PubMed, MEDLINE, Central, and Google Scholar up to that date. The search targeted studies published in English, French, Portuguese, Italian, or Spanish, utilizing MeSH terms such as [“peritoneal dialysis” AND “glucose-based dialysate”], [“peritoneal dialysis” AND “Glucose sparing”], and [“peritoneal dialysis AND “glucose-based dialysate” AND “Complications”].

Additionally, a free-text search of titles and abstracts included terms such as kidney failure, peritoneal dialysis, hemodialysis, renal replacement therapy, and other relevant items in different combinations helped to identify relevant literature. Reference lists of identified studies were hand-searched, and domain experts recommended key articles. Eligible publications included systematic reviews, meta-analyses, randomized controlled trials, observational studies (cohort, cross-sectional, case-control), and case series. This approach ensured the inclusion of diverse study designs to capture a broad perspective on the impact of glucose-based and glucose-sparing dialysates in peritoneal dialysis.

During the process of manuscript writing and discussions between experts, additional relevant articles were identified and added as references.

## Glucose-based dialysate: implications for glycemic control and metabolic status

Glucose is the predominant osmotic agent in PD solutions in clinical practice, containing, in most cases, depending on the manufacturer, either dextrose monohydrate concentrations of 1.5%, 2.5%, and 4.25%, corresponding to anhydrous glucose levels of 1,360, 2,270, and 3,860 mg/dL, i.e., 1.36%, 2.27%, and 3.86%, respectively, or anhydrous glucose concentrations 1.5%, 2.3% or 4.25% (Table 1).

**Table 1:** Composition of common commercially available peritoneal dialysis solutions

Component (mmol/L)	Dianeal PD4/PD2^®^	Stay-Safe^®^	Physioneal 35^®^	Physioneal 40 ^®^	Balance^®^	BicaVera^®^	Nutrineal^®^	Extraneal^®^
Sodium	132	134	132	132	134	134	132	132
Calcium	1.25	1.25/1.75	1.75	1.25	1.25/1.75^a^	1.25/1.75	1.25	1.75
Magnesium	0.25/1.75	0.50	0.25	0.25	0.50	0.50	0.25	0.25
Chloride	95/96	102.5/103.5	101	95	100.5/101.5	103.5	105	96
Lactate	40	35	10	15	35	0	40	40
Bicarbonate	—	—	25	25	—	34	—	—
pH	5.2–5.5	pH 5.5	7.4	7.4	7.4	7.4	6.7	5.5
**Osmotic Agent**	**Glucose** ^**#**^	**Glucose**	**Glucose** ^**#**^	**Glucose** ^**#**^	**Glucose**	**Glucose**	**Amino Acids**	**Icodextrin**
**Osmotic agent** **(% w/v / gL)**	1.36%, 2.27%, 3.86%	1.5% 2.3% 4.25%	1.36%/13.6 2.27%/22.7 3.86%/38.6	1.36%/13.6 2.27%/22.7 3.86%/38.6	1.5% 2.3% 4.25%	1.5% 2.3% 4.25%	1.1%/11.1	7.5%/75
**Osmotic agent** **(mmol/L)**	75.5 126 214	83.2 126.1 235.8	75.5 126 214	75.5 126 214	83.2 126.1 235.8	83.25 126.1 235.9	87.2	4.7^b^
**Osmolarity (mOsm/L)**	344/346 395/395 483/485	356/358 399/401 509/511	345 396 484	344 395 483	356/358 399/401 509/511	356/358^c^ 399/401^d^ 509/511^e^	365	284

Sources: The composition of peritoneal dialysis solutions is from summaries of product characteristics from respective manufacturers, Vantive (former Baxter) or Fresenius Medical Care

# Anhydrous glucose; ^a^ 1.75 mmol/L calcium is not available for the 4.25% glucose solution; ^b^assuming an average molecular weight of icodextrin of 16,000 g/mol, the concentration of icodextrin in Extraneal PD solution is approximately 0.0047 mol/L, 4.7 mmol/L; ^c^Glucose 1.5% and calcium 1.25 mmol/L; ^d^Glucose 2.3% and calcium 1.75 mmol/L; ^e^Glucose 4.25% and calcium 1.75 mmol/L

These solutions facilitate UF by generating an osmotic gradient across the peritoneal membrane. Solute clearance on PD depends on diffusion and convection. Convective clearance (dependent on net UF) makes a strong contribution to overall clearance, especially of large molecules. Convection and net UF are centrally important to salt and water homeostasis in PD patients without well-preserved urine output.

However, glucose absorption into systemic circulation is inevitable, driven by the concentration gradient. Absorption rates vary with dwell time, solution volume, and membrane transport properties [17, 30–34].

### Glucose absorption and caloric load

Glucose’s small molecular size facilitates its rapid systemic absorption, diminishing the osmotic gradient and contributing to a net daily glucose uptake that may vary from less than 50 g to more than 200 g [32] depending on glucose concentration in blood and dialysate, volume, number and frequency of exchanges of dialysis fluid, and PM characteristics [35–38]. Approximately 75% of the glucose instilled is absorbed over a 6-hour dwell, with 50% occurring within the first 90 min [32]. Glucose absorption for a 6-hour dwell using 2 L of dialysate ranges from 15 to 22 g with a 1.5% solution to 46–60 g with a 4.25% solution [31]. Caloric intake from glucose absorption corresponds to 4–13 kcal/kg/day [39, 40]. Predictive tools, including kinetic modeling programs, offer personalized estimates of glucose absorption and caloric intake from PD [41, 42].

The substantial peritoneal energy intake contributes to metabolic disturbances including gains in body weight that sometimes impede the listing and eligibility for kidney transplantation [43, 44].

### Hyperglycemia and carbohydrate burden in PD

Unlike oral glucose intake, PD-associated glucose absorption prolongs hyperglycemia [45]. In a study of non-diabetic Chinese patients, 8.3% had fasting glucose levels exceeding 200 mg/dL after one month of PD, and 19.6% experienced elevated fasting glucose, demonstrating the glycemic effects of sustained glucose exposure [46]. Furthermore, patients undergoing PD with glucose-based solutions have an increased risk of developing new-onset diabetes [18]. An important underpinning mechanism may be that glucose-based PD solutions are thought to exacerbate insulin resistance, due to the continuous absorption of glucose from the peritoneal cavity [47]. Continuous glucose monitoring in diabetic CAPD patients revealed worsened glycemic control with standard glucose-based dialysates compared to glucose-sparing alternatives [48] and a loss of physiological nighttime glucose dipping in patients treated with automated PD, APD [49].

### Clinical implications and long-term impact

Although glucose-induced hyperglycemia is well recognized, its long-term impact on patients undergoing PD remains debated. Initiating PD may initially improve insulin sensitivity by alleviating uremia, partially mitigating the glucose burden [50]. Epidemiological data and meta-analyses suggest PD does not significantly increase the risk of new-onset hyperglycemia compared to hemodialysis [51, 52]. However, small-scale physiological studies consistently demonstrate elevated plasma glucose with glucose-based PD solutions in both diabetic and non-diabetic populations [48, 53–56].

The Global Fluid Study highlighted the positive association between glucose exposure and elevated random glucose levels, reflecting the metabolic demands of PD [56].

Given the complex metabolic milieu in dialysis patients, characterized by insulin resistance and multiple hyperglycemia risk factors, the precise impact of peritoneal glucose absorption on the deterioration of long-term glycemic control remains uncertain [57]. These findings underscore the urgent need for glucose-sparing strategies and alternative osmotic agents to reduce carbohydrate overload and mitigate glucose-related metabolic complications.

### Glucose exposure, oxidative stress, and cardiovascular risk

Chronic hyperglycemia initiates a cascade of metabolic disruptions that severely compromise vascular function [58–62]. Prolonged elevation of glucose concentrations promotes the formation of advanced glycation end products (AGEs), which bind to their receptor, RAGE, on endothelial cells. This receptor-ligand interaction activates pro-inflammatory signaling pathways, thereby inducing oxidative stress (OS) and promoting vascular remodeling [58–62]. These molecular events significantly impair endothelial function, laying the groundwork for endothelial dysfunction, a pivotal precursor to atherosclerosis and other cardiovascular complications [62, 63].

Hyperglycemia further amplifies OS by increasing reactive oxygen species (ROS) production while concurrently diminishing antioxidant defenses. The resultant ROS accumulation drives lipid peroxidation, protein oxidation, and deoxyribonucleic acid (DNA) damage, all of which compromise endothelial integrity [64]. The oxidative deterioration of vascular components not only impairs vascular reactivity but also escalates inflammation, hastening the progression of atherosclerosis [61, 62].

Lipid peroxidation, a key consequence of ROS activity, disrupts membrane stability, while protein fragmentation and DNA oxidation impair cellular functions [65]. Specifically, hydroxyl radicals initiate lipid oxidation by abstracting hydrogen ions, generating lipid radicals that perpetuate further oxidative chain reactions. This cascade yields malondialdehyde (MDA), a critical mediator of atherogenesis [66]. The cumulative oxidative damage induces endothelial dysfunction, systemic inflammation, and atherosclerosis, thus intensifying the link between cardiovascular and renal disease [67]. This interconnected cycle involving OS, inflammation, and endothelial dysfunction substantially contributes to the elevated cardiovascular morbidity and mortality in patients with ESKD [67].

In chronic dialysis patients, OS is exacerbated by nicotinamide adenine dinucleotide phosphate (NADPH) oxidase activation and RhoA/Rho kinase (ROCK) signaling, pathways implicated in cardiovascular pathology. Inhibiting ROCK signaling is associated with cardioprotective effects [67, 68].

In the context of PD, OS primarily arises from AGEs and glucose-derived pro-oxidants [69]. GDPs, generated during heat sterilization of glucose-based dialysates, accumulate and further promote AGE formation [70].

Recent molecular biology studies have confirmed exacerbated OS in PD patients, as demonstrated by increased OS markers. Elevated levels of p22phox, MYPT1 activity (a Rho kinase signaling marker), and ferritin were observed, with further increases recorded after six months of PD therapy [71].

The oxidative burden in PD is further amplified by the properties of conventional PD solutions with high glucose concentration, elevated osmolarity, and acidic pH—factors that render them non-physiological and harmful to cellular homeostasis [70, 71].

Additionally, glucose in PD solutions influences lipid metabolism, potentially elevating triglyceride (TG) and total cholesterol (TC) levels [72, 73]. The association between an atherogenic lipid profile induced by high-glucose dialysates and increased cardiovascular disease (CVD) risk is well-documented [74]. Wen et al. reported a correlation between higher peritoneal glucose concentrations and increased all-cause and CVD mortality [75]. However, findings by Law et al. indicated no significant relationship between glucose absorption and serum lipid profiles after adjusting for confounders [76].

The relationship between CVD, mortality, and lipid alterations in PD patients remains complex and contentious. A phenomenon of reverse epidemiology has been observed, where lower cholesterol levels and lower BMI paradoxically correspond with higher mortality rates in PD populations [77–79]. It is thought that malnutrition and inflammation present greater cardiovascular risks than dyslipidemia [80]. Wang et al. recently identified a positive relationship between glucose absorption and lipid profiles; however, increased glucose absorption was linked to lower CVD risk in patients with reduced protein intake, but elevated risk in those with higher high-sensitivity C-reactive protein (hs-CRP) or greater protein consumption [81].

These findings underscore the need for more biocompatible dialysis solutions, including those formulated with amino acids or other non-glucose alternatives, to mitigate OS and inflammatory injury in PD patients.

### Glucose-based solutions and peritoneal membrane: oxidative stress and structural damage in long-term peritoneal dialysis

The prolonged use of glucose-based PD solutions leads to cumulative structural and functional alterations of PM [19, 69, 70, 82–85]. Exposure of PM cells to high-glucose dialysates intensifies AGE accumulation, eliciting oxidative and inflammatory responses that contribute to peritoneal tissue damage [70] and the continuous exposure to non-biocompatible PD solutions initiates progressive damage [19, 82–86]. High glucose content, GDPs, and AGEs induce OS, a major pathological driver of PM injury [87]. Chronic exposure leads to fibrosis, vasculopathy, neo-angiogenesis, and mesothelial cell (MC) transformation, impairing dialysis efficacy and leading to adverse patient outcomes [88–90]. Clinical consequences of these changes can include encapsulating peritoneal sclerosis.

#### Structural changes of the peritoneal membrane

The PM consists of a monolayer of MCs adhered to a basement membrane, beneath which lies the submesothelial layer containing fibroblasts and blood vessels [86]. According to the three-pore model of peritoneal transport, the main routes or barriers for solute and fluid transport through the PM are the ultrasmall transcellular pores (aquaporins) of the endothelial cells, and the small and large pores in between the endothelial cells of the capillary walls. Exposure to hyperosmotic and bioincompatible solutions initiates histologic alterations, including microvilli loss, cellular hypertrophy, and mesothelial cell detachment. The thickened submesothelial zone, combined with altered solute transport and reduced UF capacity, marks the progression to peritoneal fibrosis [91].

#### Epithelial -to-mesenchymal transition (EMT)

Prolonged mesothelial injury promotes EMT, where epithelial-like mesothelial cells acquire mesenchymal characteristics, enhancing motility and extracellular matrix secretion [92]. This reversible process involves loss of cellular polarity and dissolution of intercellular junctions. Downregulation of epithelial markers, such as E-cadherin, occurs due to Snail induction, while tight junction proteins, including claudin and occludin, are disrupted [93]. Cytokines, inflammatory factors, and transcription regulators orchestrate EMT through the transforming growth factor-beta (TGF-β) pathway. TGF-β1 triggers Smad-dependent and non-Smad signaling, regulating fibrosis-associated genes such as Snail, alpha-smooth muscle actin (α-SMA), and collagen [93, 94].

#### Functional changes of the peritoneal membrane

Glucose-based PD solutions have been associated with functional impairment of the PM over long-term use, primarily through the loss of UF capacity [36]. The BalANZ Study, the largest randomized trial examining the effect of biocompatible solutions on membrane function, showed that initial PSTR was faster with biocompatible solutions compared to standard solutions [95]. Notably, over a two-year period, PSTR remained stable in patients treated with biocompatible solutions, while it increased in those receiving standard glucose-based solutions, indicating a slower deterioration in membrane function with biocompatible alternatives [95]. Several studies have demonstrated a relationship between PSTR and adverse health outcomes, including mortality and PD technique failure [96].

A secondary analysis of the BalANZ trial revealed that peritoneal GDP exposure may be a more important consideration in preserving peritoneal membrane function over time than peritoneal glucose exposure [97].

Three meta-analyses comparing neutral-pH, low-GDP, and conventional solutions have been published by Cho et al. [20], Seo et al. [98], and Yohanna et al. [99]. These studies indicate that treatment durations longer than 6 months with neutral-pH, low-GDP solutions, as compared to conventional PD solutions, are associated with enhanced RKF.

On the other hand, Cho et al. [100] found that, in comparison to PD solutions with high-GDP levels, those with low-GDP levels exhibited a reduced UF volume during the peritoneal equilibration test, as well as a lower daily UF volume during the first year following the initiation of peritoneal dialysis.

##### Glucose-induced pseudohypoxia

Continuous exposure of the peritoneum to high-glucose dialysis solutions causes pseudohypoxia. This causes increased expression of hypoxia-inducible factor-1 (HIF-1) by interstitial cells, leading to increased expression of glucose transporter type 1 (GLUT-1) and profibrotic factors (TGFb, vascular endothelial growth factor [VEGF], plasminogen activator inhibitor-1 [PAI-1], and connective tissue growth factor [CTGF]). Compensatory mechanisms may be impaired in PD due to mitochondrial dysfunction and the use of lactate as a buffer in PD solutions [101].

Glucose-induced pseudohypoxia is likely a key driver of long-term peritoneal alterations. This condition mimics true hypoxia by increasing the intracellular reduced and oxidized nicotinamide adenine dinucleotide (NADH/NAD⁺) ratio, thereby disrupting cellular redox homeostasis. Pseudohypoxia activates the transcription factor HIF-1, which upregulates fibrotic mediators such as TGF-β, CTGF, PAI-1, and GLUT-1 [102]. These changes contribute to interstitial fibrosis and to a progressive decline in peritoneal free water transport (FWT) in long-term PD treatment, and peritoneal thickening [103]. The association between pseudohypoxia and upregulation of CD24 further supports its central role in peritoneal remodeling during long-term PD [104, 105].

#### Oxidative stress in peritoneal membrane injury

High-glucose PD solutions exacerbate ROS production, overwhelming antioxidant systems, and damaging mitochondrial DNA. GDPs and AGEs activate RAGE, amplifying ROS-driven pro-inflammatory and fibrotic cascades [106]. Elevated nuclear factor kappa-light-chain-enhancer of activated B cells (NF-κB) and VEGF levels induce PM thickening, fibrosis, and angiogenesis. Mesothelial apoptosis exceeds 60% within two hours of exposure to 4.25% glucose solutions, indicating rapid cellular damage [107].

#### Cytokine and chemokine pathways

OS-induced cytokine production by MCs, including interleukin-1 (IL-1), IL-6, and IL-8, activates inflammatory signaling. IL-6 and IL-8 initiate Janus kinase (JAK)/signal transducer and activator of transcription (STAT) pathways, promoting mesenchymal marker deposition [108]. TGF-β1 signaling further enhances EMT and fibrosis [93, 109]. Chronic inflammation stimulates neo-angiogenesis, expanding the PM surface area for solute transport. VEGF, upregulated by TGF-β and pro-inflammatory cytokines, drives vascular changes. Reduced VEGF levels after switching to glucose-free solutions demonstrate the glucose-dependency of VEGF expression [93].

#### Macrophage polarization and inflammation

Persistent glucose exposure induces macrophage polarization, favoring M2 macrophages via the Arginase 1 pathway. This shift contributes to EMT, fibrosis, and impaired repair. High-glucose environments suppress M1 macrophages through microsomal prostaglandin E synthase-1 activation, exacerbating extracellular matrix synthesis. Autophagy, a compensatory mechanism to mitigate ROS, may fail under prolonged stress, causing lysosomal dysfunction, apoptosis, and intensified inflammation [110, 111].

**Fig. 1:**
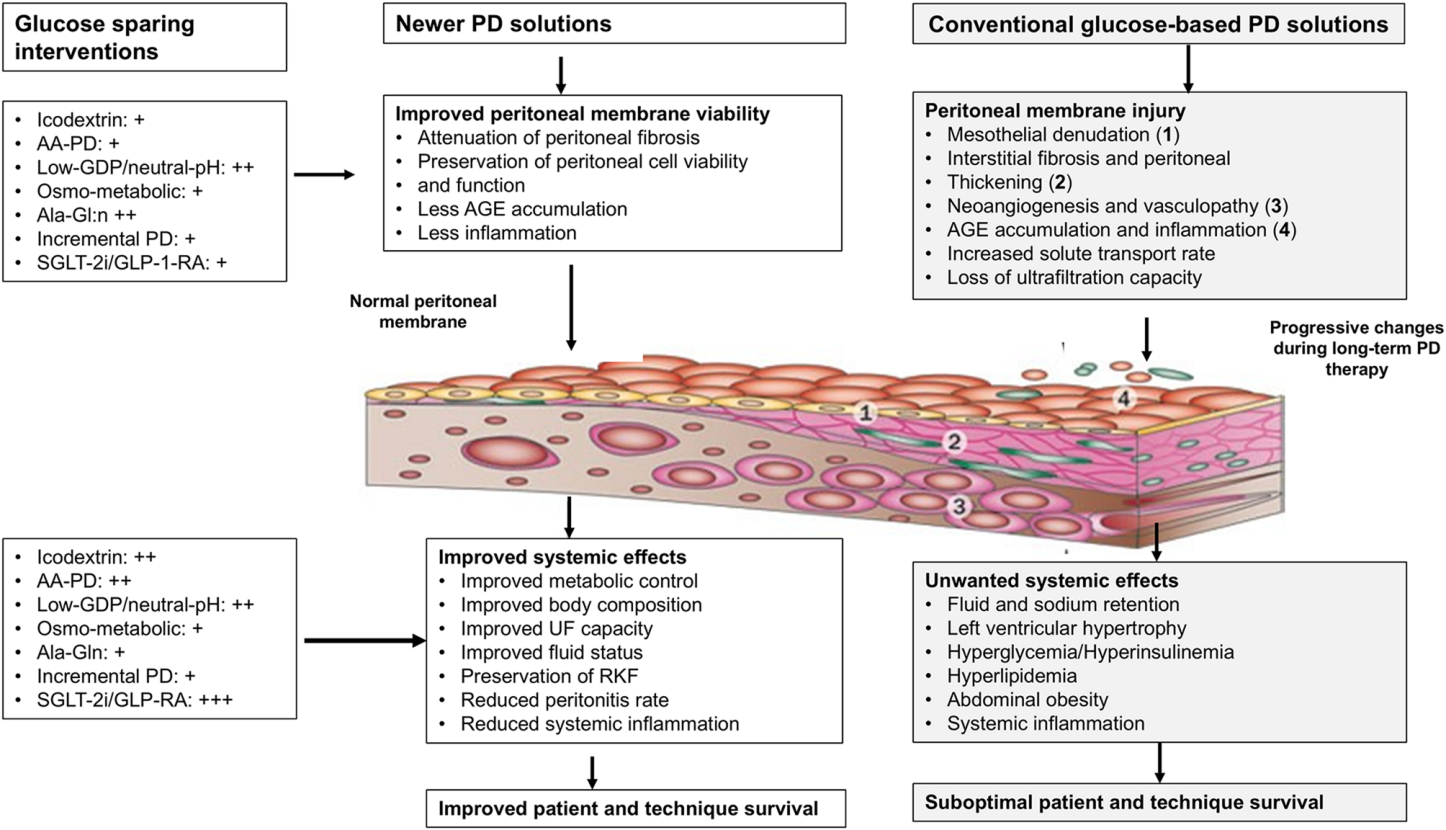
Schematic representation of proposed beneficial effects of novel peritoneal dialysis solutions. Reproduced with permission of the copyright owner from García-López E et al. (Reference [27]) and examples of glucose sparing PD solutions and other interventions. Abbreviations: AGE: Advanced glycation end product; PD: Peritoneal dialysis; RKF: Residual kidney function; UF: Ultrafiltration; AA-PD: amino acid-based PD solution; GDP: glucose degradation product; Ala-Gln: Alanyl-Glutamine PD solution supplemented with the dipeptide alanyl-glutamine; SGLT-2i: sodium-glucose cotransporter-2 inhibitors; GLP-1 RA: glucagon-like peptide-1 receptor agonists. +, ++ and +++ denote potential estimated positive local/peritoneal or systemic effects of glucose sparing interventions

Figure 1 provides an overview of the local and systemic effects induced using conventional hyperglycemic PD solutions.

#### Bioincompatible solutions and pH effects

The acidic pH of conventional PD solutions aggravates oxidative damage. Acidic environments promote the release of free iron from transferrin, driving lipid peroxidation and protein carbonylation. Neutral pH, low GDP solutions reduce OS and enhance mesothelial viability. Bicarbonate-based buffers outperform lactate buffers in preserving PM integrity [112–114]. Animal models demonstrate diminished fibrosis and angiogenesis with improved buffer composition [114].

Both pH and lactate are also implicated in alterations of peritoneal defenses, such as decreased phagocytic capacity of macrophages, leading to intracellular acidification and thus altered neutrophil function. On the other hand, both pH and lactate increase mesothelial cytotoxicity and the adverse effects of these solutions. In PD, compensatory mechanisms may be compromised because of mitochondrial dysfunction and the reliance on lactate as the buffering agent in dialysis solutions [113].

In conclusion, OS is central to PM injury in PD, driven by hyperglycemia, GDPs, and AGEs. Effective mitigation requires enhanced biocompatible solutions and antioxidant therapies. Innovations targeting oxidative pathways are imperative to sustain long-term dialysis efficacy and patient health.

## Metabolic and therapeutic strategies for preserving peritoneal membrane function and mitigating fibrosis in peritoneal dialysis

### Metabolic modulation as an antifibrotic strategy

Targeting glycolysis, fatty acid oxidation, and pyruvate metabolism offers additional avenues for fibrosis prevention. Pharmacological activation of carnitine palmitoyl transferase 1 (CPT1) or suppression of malonyl-CoA via malonyl-CoA decarboxylase (MCD) reduces glycolytic reliance. Alternatively, inhibiting pyruvate dehydrogenase kinase isoenzyme 1 (PDK1) with dichloroacetate (DCA) activates pyruvate dehydrogenase (PDH), channeling pyruvate into the tricarboxylic acid cycle instead of lactate production, mitigating extracellular matrix acidification [115–117]. Direct glycolytic inhibition using 2-deoxyglucose, a hexokinase 2 inhibitor, effectively disrupts glycolysis and decreases TGF-β1-induced fibrotic phenotypes in mesothelial cells [118, 119].

The switch from oxidative phosphorylation to glycolysis, driven by TGF-β1, highlights the Warburg effect’s role in EMT and fibrosis. Attenuating glycolytic flux with 2-deoxyglucose substantially reduced glucose-induced fibrosis in animal models, though the relative safety of glycolysis inhibition versus promoting oxidative metabolism requires further investigation [119–121].

### Therapeutic strategies for OS mitigation

Biocompatible solutions incorporating low GDPs, neutral pH, and alternative osmotic agents like icodextrin offer some OS protection. Antioxidants such as N-acetylcysteine (NAC), pyruvate, and alanyl-glutamine (AlaGln) are promising in ROS reduction. NAC scavenges ROS, preventing lipid peroxidation and DNA fragmentation. Pyruvate buffers hydrogen peroxide, preserving mitochondrial function, while AlaGln restores mesothelial defenses and reduces OS markers [122–126].

Managing PM fibrosis in PD requires a multifaceted approach that addresses both metabolic and OS pathways. Interventions targeting fatty acid oxidation, pyruvate oxidation, and glycolysis, alongside strategies to modulate extracellular factors such as TGF-β, VEGF, and inflammatory cytokines, are crucial. Additionally, intracellular mediators like HIF-1α should be considered in the development of biocompatible PD solutions. OS, which plays a central role in PM injury, is driven by factors such as hyperglycemia, GDPs, and AGEs [70, 93, 127]. To mitigate these effects, enhanced biocompatible solutions and antioxidant therapies are necessary. Targeting oxidative pathways is essential for the design of effective antifibrotic therapies aimed at sustaining long-term dialysis efficacy and improving patient health.

Other approaches targeting oxidative pathways and fibrosis have included AlaGln-supplemented PD fluid which was reported to improve biomarkers of PM integrity, immune competence, and systemic inflammation compared to not supplemented PD fluid with neutral pH and low GDPs [125]. The same group have also reported that addition of lithium chloride to the PD solution could counteract mesothelial cell death, peritoneal membrane fibrosis, and angiogenesis [128].

## Innovative alternatives to conventional glucose-based peritoneal dialysis solutions: biocompatibility and metabolic impact

Glucose remains the predominant osmotic agent in conventional PD solutions due to its cost-effectiveness, safety, and efficient UF properties. However, elevated glucose concentrations in PD solutions result in increased absorption, leading to metabolic issues such as hyperglycemia, hyperinsulinemia, obesity, and hyperlipidemia [129]. As a result, non-glucose-based osmotic agents such as icodextrin and amino acids are increasingly used in glucose-sparing regimens to mitigate these metabolic side effects. Table 1 shows an overview of the composition of some common commercially available PD solutions, while Table 2 summarizes the main characteristics of current and new PD solutions.

**Table 2:** Key characteristics and in vivo advantages and disadvantages of different peritoneal dialysis solutions. Adapted from Bonomini et al. [84], Low & Liew [130], and Bonomini et al. [131]

Peritoneal dialysis solution	Glucose load	Glucose sparing	GDP formation	Systemic potential benefit	Peritoneal potential benefit	Osmo-metabolic benefits*
Glucose-Based Lactate Buffer	High exposure	No	High production	Nutritional	Osmotic	No
Biocompatible Glucose-Based Lactate and/or Bicarbonate Buffer	High exposure	No	Low production	Nutritional	Osmotic and pH modulation	No
Icodextrin	None	Yes	Minimal formation	Volume regulation Uremic toxins clearance Metabolic control	Long-dwell ultrafiltration (UF)	Yes
Amino Acids	None	Yes	None	Protein synthesis	Osmotic	Yes
Glycerol and Amino Acids	None	Yes	None	Nutritional	Osmotic	Yes
Xylitol–Carnitine–Glucose	Reduced exposure	Yes	Moderate production	Antidiabetic	Osmotic, antifibrotic, and antiangiogenic	Yes
Glucose and Carnitine	Conventional Exposure	No	Moderate production	Carnitine deficiency	Osmotic and membrane preservation	Yes
Glucose and Alanyl-Glutamine	Conventional Exposure	No	Moderate production	Reduced protein loss	Osmotic and membrane preservation	No
Glucose and Sulodexide	Conventional Exposure	No	Moderate production	Anti-inflammatory	Osmotic and enhanced dialysis efficiency	No

*Osmo-metabolic benefits: PD solutions demonstrate osmotic and metabolic effects. GDP: Glucose degradation products; UF: Ultrafiltration

Icodextrin, a glucose polymer derived from starch, has been shown to induce a slower but sustained UF rate, offering improved fluid balance and blood pressure regulation [132], with potential benefits for left ventricular mass reduction [133]. Furthermore, icodextrin´s effects on glucose metabolism have been favorable in clinical trials [21] and real-world studies [134], and it may prolong the survival of ESKD patients on PD [135].

Amino-acid-based PD solutions, like Nutrineal^®^, present an alternative with no glucose content and may when used together with icodextrin replace up to 50% of the daily glucose load [83, 84]. These solutions are particularly beneficial for malnourished PD patients, as they improve nitrogen balance and nutritional markers such as albumin and other plasma proteins [136, 137]. However, despite these improvements, no clear mortality benefit has been observed. The biocompatibility of amino-acid-based solutions remains debated, with studies showing preservation of peritoneal UF and reduced sub-mesothelial fibrosis in animal models [138], although concerns about nitric oxide generation have been raised [139].

The clinical impact of the combined use of low-GDP glucose-based solutions, icodextrin, and amino-acid based solutions (Physioneal, Extraneal and Nutrineal, PEN or NEPP) has been explored in both APD and CAPD patients. The IMPENDIA study investigated the effects of such a glucose-sparing PD regimen in diabetic patients [140]. The findings indicated that substituting icodextrin and amino acid–based dialysis fluids for glucose-based solutions in two daily PD exchanges leads to a reduction in HbA1c levels. Additionally, this approach resulted in moderate yet significant improvements in lipid parameters, including reductions in triglycerides, very-low-density lipoprotein (VLDL), and apolipoprotein B levels [140]. However, the study also identified a higher incidence of severe adverse events, such as mortality and heart failure, in the glucose-sparing cohort. These results suggest that while a low-glucose PD regimen enhances metabolic outcomes, it may elevate the risk of extracellular fluid volume overload if patients are not adhering to fluid intake restrictions. It should be noted that there are non-responders to icodextrin with respect to UF [141]. Therefore, careful monitoring of fluid balance is essential when implementing glucose-sparing dialysis strategies [140].

The recently published DiDo study is a randomized control trial that demonstrated that the use of two icodextrin bags per day is safe, significantly increases ultrafiltration, and concurrently reduces glucose exposure [24].

Improvement of nutritional status by increased synthesis of proteins is only achieved if enough calories (carbohydrates) are ingested simultaneously [142], and higher incidence of adverse events may occur due to disregard of these aspects.

The development of neutral-pH, low-GDP PD solutions, containing lactate and/or bicarbonate buffers, which aim to reduce the bioincompatibility of conventional glucose-based dialysis fluid have shown benefits in preserving PM integrity, and in some studies preservation of RKF [7, 20, 99, 130, 131, 143].

L-carnitine, through its role in fatty acid oxidation and pyruvate metabolism, can be used in carnitine-enriched PD solutions to mitigate PM fibrosis by enhancing pyruvate oxidation and reducing myofibroblast activation in patients with ESKD [21, 144, 145], see Table 2.

Used for over 50 years in food, cosmetic, and pharmaceutical industries, endogenously produced sugar alcohol sweeteners erythritol and xylitol minimally affect plasma glucose and insulin levels while promoting the release of beneficial gastrointestinal hormones, such as e.g., glucagon-like peptide-1 [146]. Xylitol (151 Da) is a five-carbon sugar alcohol naturally produced in humans through D-xylulose reduction and is approved as a glucose substitute for parenteral nutrition in some countries [21]. Intravenous administration does not induce hyperglycemia, leads to lower insulin secretion than glucose, and is primarily metabolized in the liver. In vitro studies suggest better biocompatibility compared to glucose, while clinical trials indicate its effectiveness as an osmotic agent and its potential to improve glycemic control in diabetic PD patients [147]. Reducing glucose exposure in PD solutions may be beneficial especially if coupled with strategies that address insulin resistance directly and reduce excessive use of insulin treatment in type 2 diabetes [148]. The use of L-carnitine and xylitol in PD solutions may contribute to an ‘osmo-metabolic approach’ to a glucose-sparing PD strategy by supporting ultrafiltration and metabolic regulation [21]. ELIXIR (ClinicalTrials.gov Identifier: NCT03994471) is an ongoing phase III, open-label, randomized controlled trial assessing Xylocore^®^, a formulation containing xylitol and L-carnitine, against conventional glucose-based regimens in patients with kidney failure undergoing CAPD [149].

While studies have demonstrated the preservation of endothelial glycocalyx and vascular function in adult patients treated with neutral-pH, low-GDP solutions [150, 151], the long-term effects on PM health are uncertain. In pediatric populations, research has indicated that despite less severe morphological changes in the PM with neutral-pH fluids, there is still evidence of peritoneal fibrosis and vascular changes [150, 152, 153]. These findings suggest that the biocompatibility of neutral-pH, low-GDP solutions is not yet fully established and warrants further investigation. While non-glucose-based PD solutions offer promising metabolic and fluid-handling benefits, the overall biocompatibility and long-term effects on peritoneal health require further study to optimize these solutions for clinical use.

### Bimodal solutions

Another approach to improve fluid removal and thereby potentially reducing the need for additional glucose-based solutions is to combine crystalloid (glucose) and colloid (icodextrin) osmotic agents to markedly enhance peritoneal fluid and solute transport during the long PD dwell [154, 155]. There are different variants of bimodal PD solutions with different combinations of icodextrin and dextrose to provide more efficient UF and sodium removal than traditional PD solutions [154–157].

### Glucose-sparing using alternative prescription patterns and drugs

Maintaining RKF is of critical importance to reduce the need for high-glucose solutions and strategies to achieve this include, in addition to glucose-free solutions, biocompatible solutions, appropriate prescriptions, and pharmacological therapies [158]. Alternative glucose-sparing strategies, in addition to or together with use of glucose-free dialysis solutions, include prescriptions such as incremental PD that may reduce glucose exposure or absorption. Furthermore, use of new classes of anti-diabetic drugs such as sodium-glucose co-transporter 2 (SGLT2) inhibitors that preserve RKF as well as peritoneal membrane structure and function may facilitate adequate fluid removal and thus diminish the need for boosting peritoneal UF by increasing the concentration of glucose in PD solutions. A brief overview of mechanisms, benefits, limitations, and evidence strengths of these and other glucose-sparing strategies in PD is provided in Table 3.

**Table 3:** Overview of mechanisms, benefits, limitations, and evidence strengths of different glucose-sparing strategies in peritoneal dialysis

Strategy / modality	Mechanism / description	Benefits	Limitations	Evidence strength
Icodextrin [21, 132–135]	Colloid osmotic agent for the long dwell.	Improved long-dwell UF, reduced glucose exposure, better fluid control.	One exchange per day (off label two), non-responders, rash, BG monitoring.	High (multiple RCTs for UF; moderate for long-term outcomes).
Amino-acid PD solutions [84, 136–139]	Amino acids as osmotic agent.	Reduced glucose load; improves nitrogen balance in malnourished patients.	Limited to one daily exchange due to nitrogen load; possible biocompatibility issues.	Moderate (small RCTs).
Low-GDP, neutral-pH glucose solutions [7, 20, 99, 130, 131, 143]	Reduced GDPs; more biocompatible formulation.	Preservation of RKF and urine volume; improved biocompatibility.	Higher cost; uncertain long-term effects on mortality.	High for RKF; moderate for other outcomes.
Bimodal / combination solutions [21, 142]	Glucose plus icodextrin combines osmotic profiles.	Enhanced UF and sodium removal; reduced glucose load.	Limited clinical trials; regulatory issues.	Low–moderate (pilot trials).
Osmo-metabolic solutions (xylitol, carnitine) [21, 84, 130, 131, 144–153]	Sugar alcohols and metabolic cofactors.	Lower glucose absorption; improved metabolic profile.	Investigational; limited safety data.	Low (ongoing trials).
Incremental PD & glucose-sparing prescriptions [154–157, 159–162]	Fewer glucose exchanges when RKF present.	Lower glucose exposure; preserves RKF; reduces peritonitis.	Risk of underdialysis if RKF declines.	Moderate (observational evidence).
SGLT2 inhibitors [158, 163–171]	Reduced peritoneal glucose uptake; systemic renal benefits.	Potential PM protection; CV/renal benefits.	Limited PD-specific data; possible adverse metabolic effects.	Low–moderate (emerging evidence)
GLP-1 RAs [172–175]	Improved glycemic and BP control; systemic organ protection	Improved metabolic control in patients with obesity and diabetes	Limited PD-specific data; possible adverse metabolic effects	Low (emerging evidence)
Additives (Ala-Gln, NAC, pyruvate) [122–126]	Cytoprotective / antioxidant additives.	Improved biomarkers of PM integrity.	Limited clinical outcomes; regulatory hurdles.	Low (mechanistic/early clinical).
Experimental metabolic modulators [115–121]	Modulate glycolysis or transport pathways.	Promising antifibrotic preclinical data.	Preclinical only; safety unknown.	Very low.

Abbreviations: BG: blood glucose; UF: Ultrafiltration; RCT: Randomized clinical trial; PD: Peritoneal dialysis; GDP: Glucose degradation products; RKF: Residual kidney function; SGLT2: Sodium-glucose co-transporter 2; GLP-1 RA: glucagon-like peptide-1 receptor agonists; BP: blood pressure; PM: Peritoneal membrane; CV: Cardiovascular; Ala-Gln: Alanyl-glutamine; NAC: N-acetylcysteine

### Incremental PD

There are many ways by which UF and solute clearance can be maintained while exposure to intraperitoneal glucose and therefore absorption from the peritoneal cavity is kept low. Incremental PD, a prescribing modality used in an increasing number of centers and facilitated by the availability of icodextrin-based PD solutions for the long dwell, may have several advantages, including glucose-sparing effects in addition to facilitating assisted PD, reduced costs [159], and reduction of the rate of peritonitis [160, 161].

In Italy, incremental PD, which was used by 35.3% of incident PD patients in 2022 with a further increase thereafter, has been accompanied by a reduction in peritonitis rate (and dropouts due to peritonitis), and a reduction in sclerosing peritonitis. While the causes may be multiple, glucose-sparing through incremental prescriptions is thought to play a role [162].

### SGL2 inhibitors and other glucose-sparing drugs

There is emerging evidence from experimental studies and post hoc analyses of randomized clinical trials that SGLT2 inhibitors are well tolerated and may also be effective in preventing cardiovascular and mortality outcomes in patients with severe chronic kidney disease, including patients receiving dialysis [163–170]. As such, extending the usage of SGLT2 inhibitors to dialysis patients could provide a major advancement in their care. Patients on PD have an additional unmet need for effective pharmacotherapy to preserve their RKF, with its associated mortality benefits, and for treatment options that help reduce the risk of transfer to hemodialysis.

Experimental data suggest that SGLT2 inhibitors, via various mechanisms, may preserve RKF and protect the peritoneal membrane [163].

The use of oral SGLT-2 inhibitors resulted in reduced glucose uptake and, thus, increased ultrafiltration through murine peritoneum; that study also proved that SGLT-2 receptors are expressed in the human peritoneum and HPMC and that glucose consumption and uptake by HPMC in conditions with high glucose concentrations have decreased with SGLT-2 [164].

Another study showed that canagliflozin inhibited the HIF-1α/TGF-β/ phospho-Smad3 signaling, prevented peritoneal fibrosis and peritoneal thickening, and improved peritoneal transportation and ultrafiltration. High glucose peritoneal dialysate increased the expression of peritoneal GLUT1, GLUT3 and SGLT2, all of which were inhibited by canagliflozin [165].

Considering that studies confirmed the expression of SGLT2 in the human peritoneum and experimental data suggest that SGLT2 inhibitors may decrease glucose absorption from the PD solution, thereby potentially increasing the UF volume, there is a strong rationale for studies evaluating effects of SGLT2 inhibitors in PD patients. Several studies have already been performed, and more are in the pipeline [163, 166]. One clinical trial - in chronic diabetic PD patients - showed that use of SGLT-2 inhibition may increase UF volume and hemoglobin levels; however, SGLT-2 inhibition was linked to subclinical metabolic acidosis [167].

Several trials aim to assess whether an SGLT2 inhibitor such as empagliflozin may increase the ultrafiltration volume in patients on PD [168].

However, according to some studies, SGLT2 inhibition may not have a glucose-sparing effect in PD. One study showed that SGLT2 inhibition does not reduce glucose absorption during experimental PD [169]. Another study showed that dapagliflozin usage in PD patients did not result in a reduction in glucose absorption across the peritoneal membrane [170].

Another substance of potential importance is phloretin. Intraperitoneal phloretin treatment reduced glucose absorption by > 30% and resulted in a > 50% higher ultrafiltration rate compared with control animals [171].

## Conclusion

Peritoneal dialysis plays a critical role in the management of ESKD and has many advantages compared with in-center hemodialysis as a life-saving kidney replacement therapy. However, its long-term effectiveness is compromised by the bioincompatibility of traditional glucose-based dialysis fluids, which induce both local and systemic toxicity. Because of the increased exposure to glucose and its byproducts, glucose-based PD solutions contribute to structural and functional changes of the PM accompanied by metabolic alterations and increased risk of cardiovascular morbidity and mortality.

Efforts to improve the biocompatibility of PD solutions have focused on reducing the unfavorable cardiometabolic effects and enhancing the preservation of PM morphology and function. Glucose-sparing strategies, such as the use of amino acid-based dialysis fluid combined with icodextrin-based solutions, are promising by offering sustained ultrafiltration without the adverse metabolic impacts associated with glucose absorption. These innovations aim to mitigate the harmful consequences of glucose exposure and may help preserve both systemic and peritoneal health. However, while initial short- and mid-term clinical studies indicate favorable safety profiles for these alternatives, long-term studies are needed to confirm their clinical efficacy and their potential to improve patient outcomes.

Moreover, adjunctive therapies targeting systemic metabolic complications, such as oxidative stress, are being explored to further enhance the therapeutic potential of PD and minimize adverse effects.

In summary, the evolution of PD therapies, driven by the development of glucose-sparing and biocompatible solutions, offers hope for improving the long-term efficacy of PD and preserving PM integrity. While progress has been made, ongoing clinical studies and further innovation are essential to address the persistent challenges in PD therapy and ensure optimal patient outcomes in the management of ESKD.

## Data Availability

The datasets used and/or analysed during the current study are available from the corresponding author on reasonable request.

## Abbreviations

AGEs: Advanced glycation end products
AlaGln: Alanyl-glutamine
APD: Automated peritoneal dialysis
α-SMA: Alpha-smooth muscle actin
BMI: Body mass index
CAPD: Continuous ambulatory peritoneal dialysis
CPT1: Carnitine palmitoyltransferase 1
CVD: Cardiovascular disease
DNA: Deoxyribonucleic acid
EMT: Epithelial -to-Mesenchymal Transition
ESKD: End-stage kidney disease
GDP: Glucose degradation products
GLUT-1: Glucose transporter type 1
CTGF: Connective tissue growth factor
DCA: Dichloroacetate
FWT: Free water transport
HD: Hemodialysis
HIF-1: Hypoxia-Inducible Factor-1
HPMC: Human peritoneal mesothelial cells
hs-CRP: High-sensitivity C-reactive protein
IL: Interleukin
JAK: Janus kinase
MC: Mesothelial cell
MCD: Malonyl-CoA via malonyl-CoA decarboxylase
MDA: Malondialdehyde
NAC: N-acetylcysteine
NADH/NAD⁺: Reduced and oxidized nicotinamide adenine dinucleotide
NADPH: Nicotinamide adenine dinucleotide phosphate
NEPP: Extraneal and Nutrineal, and Physioneal
NF-κB: Nuclear factor kappa-light-chain-enhancer of activated B cells
OS: Oxidative stress
PAI-1: Plasminogen Activator Inhibitor-1
PD: Peritoneal dialysis
PDH: Pyruvate dehydrogenase
PDK1: Pyruvate dehydrogenase kinase, isoenzyme 1
PEN: Physioneal, Extraneal. and Nutrineal
PM: Peritoneal membrane
PSTR: Peritoneal solute transfer rate
RAGE: Receptor of advanced glycation end products
RKF: Residual kidney function
ROCK: RhoA/Rho kinase
ROS: Reactive oxygen species
SGLT2: Sodium-glucose co-transporter 2
STAT: Signal transducer and activator of transcription
TC: Total cholesterol
TG: Trigliceride
TGF-β: Transforming growth factor-beta
UF: Ultrafiltration
